# Activation of the Liver X Receptor by Agonist TO901317 Improves Hepatic Insulin Resistance via Suppressing Reactive Oxygen Species and JNK Pathway

**DOI:** 10.1371/journal.pone.0124778

**Published:** 2015-04-24

**Authors:** Ying Dong, Guirong Gao, Hongyan Fan, Shengxian Li, Xuhang Li, Wei Liu

**Affiliations:** 1 Department of Endocrinology, Renji Hospital, School of Medicine, Shanghai Jiaotong University, Shanghai, China; 2 Department of Medicine/GI Division, Johns Hopkins University School of Medicine, Baltimore, United States of America; Northeast Ohio Medical University, UNITED STATES

## Abstract

Activation of Liver X receptors (LXRs), key transcriptional regulators of glucose metabolism, normalizes glycemia and improves insulin sensitivity in rodent models with insulin resistance. However, the molecular mechanism is unclear. This study is aimed to elucidate the mechanism of LXRs-mediated liver glucose metabolic regulation *in vitro* and *in vivo*. *Db/db* mice were used as an *in vivo* model of diabetes; palmitate (PA)-stimulated HepG2 cells were used as an *in vitro* cell model with impairment of insulin signaling. TO901317 (TO) was chosen as the LXRs agonist. We demonstrated that TO treatment for 14 days potently improved the hepatic glucose metabolism in *db/db* mice, including fasting blood glucose, fasting insulin level, and HOMA-IR. TO had no effect on the glucose metabolism in normal WT mice. TO-mediated activation of hepatic LXRs led to strong inhibition of ROS production accompanied by inactivation of JNK pathway and re-activation of Akt pathway. TO also suppressed the expression of gluconeogenic genes such as PEPCK and G-6-pase in *db/db* mice, but not in WT mice. In HepG2 cells, TO almost completely restored PA-induced Akt inactivation, and suppressed PA-stimulated ROS production and JNK activation. Interestingly, basal level of ROS was also inhibited by TO in HepG2 cells. TO significantly inhibited PA-stimulated expressions of gluconeogenic genes. Finally, we found that anti-oxidative genes, such as Nrf2, were up-regulated after LXRs activation by TO. These results strongly support the notion that activation of LXRs is critical in suppression of liver gluconeogenesis and improvement of insulin sensitivity in diabetic individuals. At molecular levels, the mode of action appears to be as fellows: under diabetic condition, ROS production is increased, JNK is activated, and Akt activity is inhibited; TO-mediated LXR activation potently inhibits ROS production, increases anti-oxidative gene expressions, suppresses JNK activation, and restores Akt activity. Our data provide new evidence to support LXRs as promising therapeutic targets for anti-diabetic drug development.

## Introduction

Insulin resistance is a characteristic feature of type 2 diabetes and plays a major role in the pathogenesis of the disease. It plays a central role in the development of many metabolic abnormalities and diseases, including obesity, dyslipidemia and cardiovascular diseases [1]. Although significant advances have been made in our understanding of the molecular mechanisms involved in insulin resistance, many questions remain [2].

As a major insulin sensitive organ, the liver plays a key role in glucose homeostasis. Liver insulin resistance will eventually result in altered metabolic gene expression and impaired glycometabolism. Liver X receptors (LXRs) are members of the nuclear receptor superfamily of ligand-activated transcription factors, which involve in almost all biological processes including metabolism, inflammation, and proliferation [3–5]. There are two isoforms of LXRs, LXRα and LXRβ, with different expression profiles in adult rodents: LXRα is mainly expressed in the liver, adipose tissue, intestine, kidney and macrophages, while LXRβ is broadly expressed [6]. Each isoform has its own specific transcriptional regulation in a specific tissue [7–9].

In the past, most studies were focused on the role of LXRs on the regulation of cholesterol and lipid metabolism. However, emerging evidence, using specific agonist TO901317, demonstrates that LXRs also play a critical role in glucose metabolism [5]. TO901317 has been demonstrated to be a highly specific activator for LXRα and LXRβ, with 2.3-fold potency to LXRα than to LXRβ, and it has no effect to other 12 nuclear receptors [10]. TO901317 has been shown to improve glucose metabolism in various animal models of insulin resistance, including both mice (*ob/ob* and *db/db*) and rats (ZDF and *fa/fa*) [11–13]. Nevertheless, TO901317 has no effect on glucose levels in *db/+* mice [13], or in the normal chow WT mice [11,12,14], suggesting the glucose modulating effects of LXR correlates with insulin resistance.

Free fatty acids (FFA) are thought to play a role in modulating insulin resistance, both *in vivo* and *in vitro* [15,16], but the mechanism involved is unclear [17]. Recently, oxidative stress has been proposed as a link between FFA and insulin resistance [18]. Overloaded FFA is one of the initial substrates leading to mitochondrial dysfunction and increased reactive oxygen species (ROS) in metabolic diseases. Increased ROS and oxidative damage were reported to occur prior to the development of insulin resistance, suggestive of a causative role of exceeded ROS production in insulin resistance [19,20]. Using cultured 3T3-L1 adipocytes, Houstis and colleagues showed that ROS production preceded insulin resistance and ROS scavenging rescues insulin sensitivity [21]. These studies all support the notion that ROS is a primary cause of insulin resistance. Activation of stress sensitive Ser/Thr kinases causes inhibitory phosphorylation of insulin signaling pathway. ROS accumulation inhibits phosphatase activity and thus promotes stress-kinase activity, providing a potential mechanism of ROS-induced insulin resistance. C-Jun N-terminal kinase (JNK) has been implicated in this process. Mice deficient for JNK1 are protected from diet-induced insulin resistance [22]. Decreased activity of liver JNK1 improves hepatic insulin sensitivity [23,24]. In addition, it was reported that endothelial progenitor cells (EPC) isolated from diabetic patients had substantially higher level of ROS than those from healthy controls [25]. TO901317 exhibited a protective effect on the functions of EPC under high glucose by inhibiting ROS while activating AMPK [26]. Taking together, these reports indicated potential cross talks among LXRs, FFA, ROS, and JNK in regulating insulin signaling pathway and glucose metabolism.

In this report, we investigated the potential molecular mechanisms of the action of LXR on FFA-induced insulin resistance, both *in vitro* and *in vivo*. We found that the LXR agonist TO901317 effectively reversed the palmitate-induced insulin resistance by decreasing the ROS production and suppressing the JNK pathway activation via LXRs in the liver.

## Materials and Methods

### Animals and treatment

Male *db/db* (C57BL/KsJ) mice (8-wk old) and their age-matched wild-type (C57BL/6) mice were purchased from the Model Animal Research Center of Nanjing University. Mice were all housed in a temperature-controlled (20–24°C) and humidity-controlled (45–55%) room illuminated daily from 7am to 7pm (12-h light, 12-h dark cycle), with free access of water and standard laboratory chow. The mice were acclimated for 1 week prior to the start of experiments. *db/db* mice and WT mice (controls) were divided into two groups (10–15 per group), controls/vehicle [dimethylsulfoxide (DMSO)-treated] and TO901317-treated, respectively. TO901317 (TO), an LXR agonist (Cayman Chemical Co., Ann Arbor, MI. 30 mg/kg/d, dissolved in DMSO) or an equal volume of DMSO (control/vehicle) was given via intraperitoneal (IP) injection daily for 2 weeks. Food intake and body weight were measured everyday from the initiation of the treatment to the last day of experiments. To sacrifice the animals, mice were first anesthetized with isoflurane followed by cervical dislocation. All animal experiments were approved by the Institutional Animal Care and Use Committee of Shanghai [SYXK (Shanghai 2009–0069)], and were conducted in accordance with the National Research Council Guide for Care and Use of Laboratory Animals [SCXK (Shanghai 2007–0005)].

### Blood and liver chemistry analysis

Blood was collected immediately after 15 minutes of insulin injection (Humulin R, Eli Lilly; 0.75U/kg for WT and 1.5U/kg for *db/db*), serum was isolated and stored at −80°C until analysis. The levels of insulin and FFA were determined enzymatically using Millipore Rat/mouse insulin 96 well plate assay kit, and BioAssay Systems EnzychormTM free fatty acid assay kit, respectively. The homeostasis model assessment of insulin resistance (HOMA-IR) was calculated as previously described [27]: HOMA-IR = Fasting glucose (mmol/L) × Fasting insulin (μU/ml) / 22.5. Plasma and hepatic malondialdehyde (MDA) was measured by MDA assay kit according to the manufacturer’s instructions (Nanjing Jiancheng bioengineering research institute, China) [28].

### Liver tissue processing

Liver tissues were collected immediately and stored for extraction and analysis of protein and mRNA, histology and the measurement of reactive oxidative species generation. A portion of the liver from each mouse was also fixed in 10% formalin for paraffin-embedding and H&E stain.

### Intraperitoneal insulin tolerance test

On day one right before drug treatment and on day 14 (the last day of treatment), *db/db* and WT mice were fasted overnight for 12 hours. Blood samples were collected from the tail vein for determination of baseline glucose values (0 minutes) before the injection of insulin (0.75U/kg for WT and 1.5U/kg for *db/db*). Additional blood samples were collected at regular intervals (15, 30, 60, 90 and 120 minutes) for glucose measurement. Blood glucose concentration was determined with a glucometer (Accu-Chek Performa; Roche Diagnostics).

### Cell culture and treatment

Human hepatocarcinoma HepG2 cells were obtained from the Cell Bank of the Chinese Academy of Science (Shanghai, China) and were cultured in minimum Eagle’s medium (Gibco) supplemented with 10% fetal bovine serum (Gibco), 100 units/ml penicillin (Invitrogen), and 0.1 mg/ml streptomycin (Invitrogen) at 37°C with humidified air and CO2 (5%). All studies were conducted using 80–90% confluent cells, which were treated, either singularly or in combinations, with TO901317 (TO; Sigma), Palmitate (PA; Sigma, Cat#: P9767), Insulin (INS; Eli Lilly) and N-acetylcysteine (NAC; Sigma) after overnight FBS deprivation. Reagents preparation and treatment procedures were summarized as the followings: PA and TO were dissolved in DMSO at a concentrations of 30 mM and 10 mM, respectively, as stocks. NAC was dissolved in sterile PBS (with pH adjusted to 7.4) at a concentration of 1 M as a stock. Stock reagents of TO, PA and NAC were diluted into the cultured medium at desired concentration, either one reagent at a time or in combinations, and then incubated the HepG2 cells for different time courses as indicated. If two or more reagents were used, they were added into medium simultaneously. The final concentrations of each reagent were: 100, 250, 500 and 750 μM for PA; 1.0 μM for TO; 2.5, 5 and 10 mM for NAC. Insulin was added into medium at various concentrations (1, 10, and 100 nM) 15 minutes before harvesting cells.

### ROS Measurement

#### ROS detection in liver tissue

Reactive oxygen species (ROS) generation within the Liver was measured by confocal microscope with *in situ* DHE stain. The sections (10 μm) of OCT-embedded liver tissues were incubated with 2 μM DHE (Sigma) at 37°C for 30 minutes in a humidified chamber protected from light, followed by 5 minutes of washing in PBS to remove non-intercalated ethidium bromide molecules. Images were obtained and analyzed with a Leica laser scanning confocal microscope (Leica TCS SP5 II, Leica Microsystems).

#### ROS detection in HepG2 cells

2,7-Dichlorodihydrofluorescein diacetate (DCFH-DA, Sigma) was used as ROS capture in the cells. It is cleaved intracellularly by nonspecific esterases to form 2,7-dichlorodihydrofluorescein (DCFH), which is further oxidized by ROS and becomes a highly fluorescent compound 2,7-dichlorofluorescein (DCF). In the present study, HepG2 cells were exposed to various drugs for the indicated times. DCFH-DA at 10 μM was incubated with cells for 20 minutes. After washing once with ice-cold PBS, cells were harvested and kept on ice for an immediate detection by flow cytometry (Becton Dickson). The average intensity of DCF stands for intracellular ROS levels.

### Quantitative real-time PCR

Total RNA was extracted using TRIzol reagent (Invitrogen, Carlsbad, CA) and complementary DNA was synthesized according to the manufacturer’s instructions (Takara, Shiga, Japan). Real-time quantitative PCR (SYBR Green) assays were performed using SYBR Premix Ex TaqTM (Takara, Shiga, Japan) in an Applied Biosystems 7300 sequence detector (Roche, Indianapolis, IN). Primers used in real-time PCR were showed in Table 1. The mRNA levels of all genes were normalized using β-actin (for mice) or GAPDH (for human) as internal control.

**Table 1:** Sequences of the primers used in real-time PCR.

GENES	SPECIES	FORWARD PRIMER	REVERSE PRIMER
**PEPCK**	Mouse	GAGAAAGCATTCAACGCCAGG	CACAGATATGCCCATCCGAGTC
**G-6-pase**	Mouse	AGCTCCGTGCCTATAATAAAGCAG	CATACGTTGGCTTTTTCTTTCCTC
**SREBP-1c**	Mouse	GAGCCACCCT TCAGCGAGGC GG	AGCAT AGGGT GGGTC AAATAG
**β-actin**	Mouse	CCTTCTACAATGAGCTGCGTG	ACAGCCTGGATAGCAACGTAC
**PEPCK**	Human	TGAGCTGTGTCAGCCTGATCAC	ACCGTCTTGCTTTCGACCTG
**G-6-pase**	Human	GAGATCATCTCCTTCGGAAGCG	TTAGTTATGCCCAGGATCAGCATG
**SREBP-1c**	Human	GGCGGGCGCAGATC	TTGTTGATAAGCTGAAGCATGTCT
**GAPDH**	Human	CCTTCTACAATGAGCTGCGTG	ACAGCCTGGATAGCAACGTAC

### SDS-PAGE and Western blot analysis

Liver tissues were homogenized in ice-cold radioimmunoprecipitation assay (RIPA P0013C, Beyotime, China) with protease inhibitor cocktail and phosphatase inhibitor cocktail (Complete, Mini, EDTA-free; PhosSTOP, Roche Applied Science, Germany). Homogenate was centrifuged at 4°C at 10,000 × g for 10 min, and the supernatant (total protein extract) was collected. Protein concentration was measured with the Bradford assay (protein assay kit; Bio-Rad Laboratories, Hercules, CA). For HepG2 cells, cells were washed three times with ice-cold PBS and homogenized in ice-cold lysis buffer [50 mM Tris-HCl (pH 7.5), 100 mM NaCl, 5 mM EDTA, 200 μM sodium orthovanadate, 1% Triton X-100] with protease inhibitor cocktail and Phosphatase inhibitor cocktail. Proteins were separated on 8% SDS polyacrylamide gels, and electrophoretically transferred to Hybond-C nitrocellulose membranes (Amersham Biosciences, UK). Subsequently membranes were blotted with blocking solution (5% skim milk in PBS containing 0.05% Tween 20) and then incubated with various primary antibodies. Each bound primary antibody was visualized with a corresponding secondary HRP-conjugated antibody (Jackson Lab) using ECL (Thermo scientific) and an image-acquiring system (LAS-4000 Mini, GE). The primary antibodies against Akt, phospho-Akt (p-Akt at Ser^473^), IRS1, phospho-IRS1 (p-IRS1 at Ser^307^), JNK, phospho-JNK (p-JNK at Thr^183^/Tyr^185^), were purchased from Cell Signaling; the anti-actin antibody was purchased from Abcam.

### Statistical analysis

The statistical analyses were carried out using SPSS 12.0 software with Student’s t-test. Statistical significance was set at p<0.05. All results are represented as mean ± SD.

## Results

### LXRs agonist TO901317 attenuates the Type 2 diabetic phenotype in *db/db* mice

**Fig 1:**
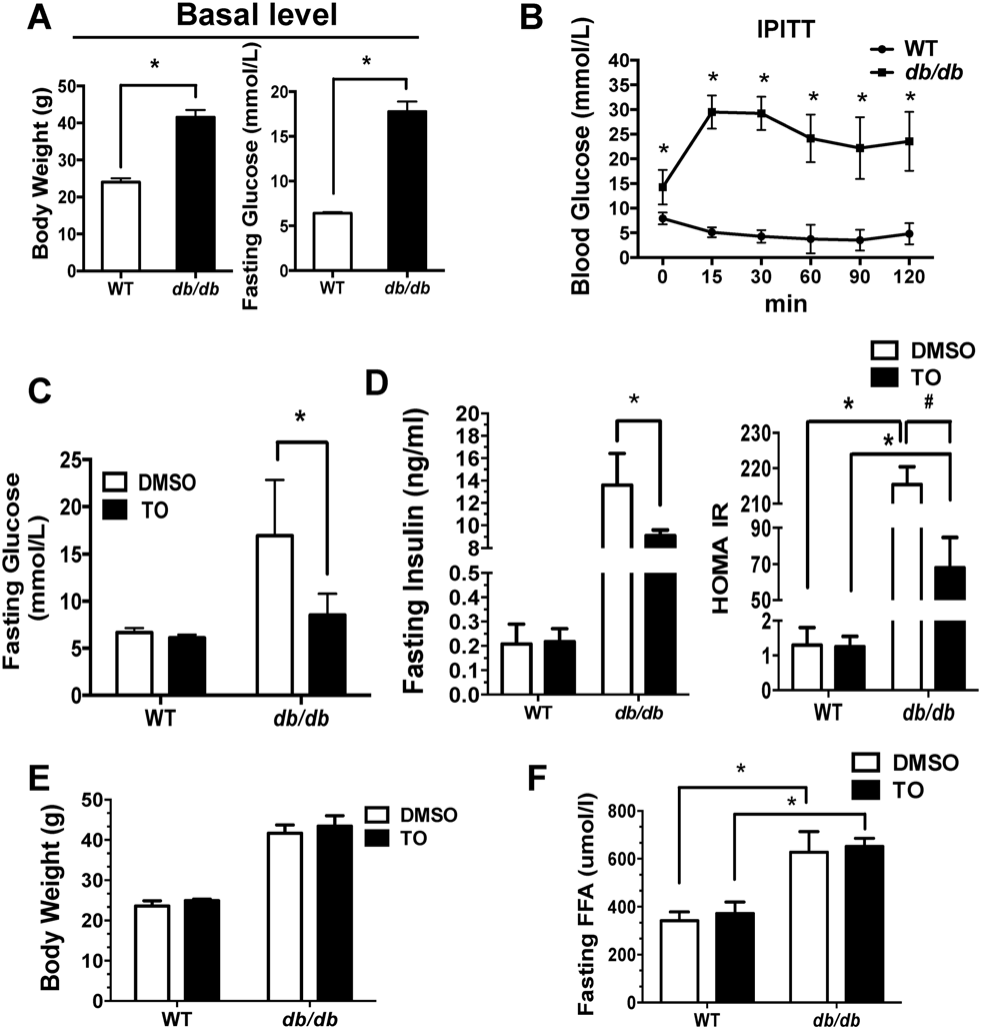
Metabolic changes in WT and *db/db* mice treated with TO901317 (TO) or DMSO (control). The WT and *db/db* mice were fed a standard laboratory diet for 9 weeks. Body weight and the fasting glucose at the basal level **(A)**, intraperitoneal insulin tolerance test (IPITT) curves before treatment **(B)** were measured. Then the mice were given a daily intraperitoneal injection of TO or DMSO respectively. Effect of TO and DMSO on fasting glucose **(C)**, fasting insulin and HOMA-IR level **(D)**, body weight **(E)** and fasting FFA **(F)** were measured after 2 weeks treatment. Results are presented as mean ± SD, *^, #^P<0.05. 10–15 mice were used in each group.

To investigate whether activation of LXRs improves the insulin sensitivity and the glucose metabolism, 9-wk-old male *db/db* mice and the age-matched WT C57/B6 mice were given a daily intraperitoneal injection of LXR agonist TO901317 (TO) or DMSO (controls) for two weeks. *Db/db* mice exhibit characteristic features of Type 2 diabetic metabolic disorders, including obesity, hyperglycemia, and insulin resistant (Fig 1A and 1B). Two-week TO treatment potently reduced the levels of fasting blood glucose, insulin, and HOMA-IR in *db/db* mice, but not in WT mice (Fig 1C and 1D). Interestingly, TO treatment has no effect on the serum levels of FFA and body weight in any experimental group (Fig 1E and 1F). In addition, food intake was similar between TO-treated group and DMSO-treated control (S1 Fig).

**Fig 2:**
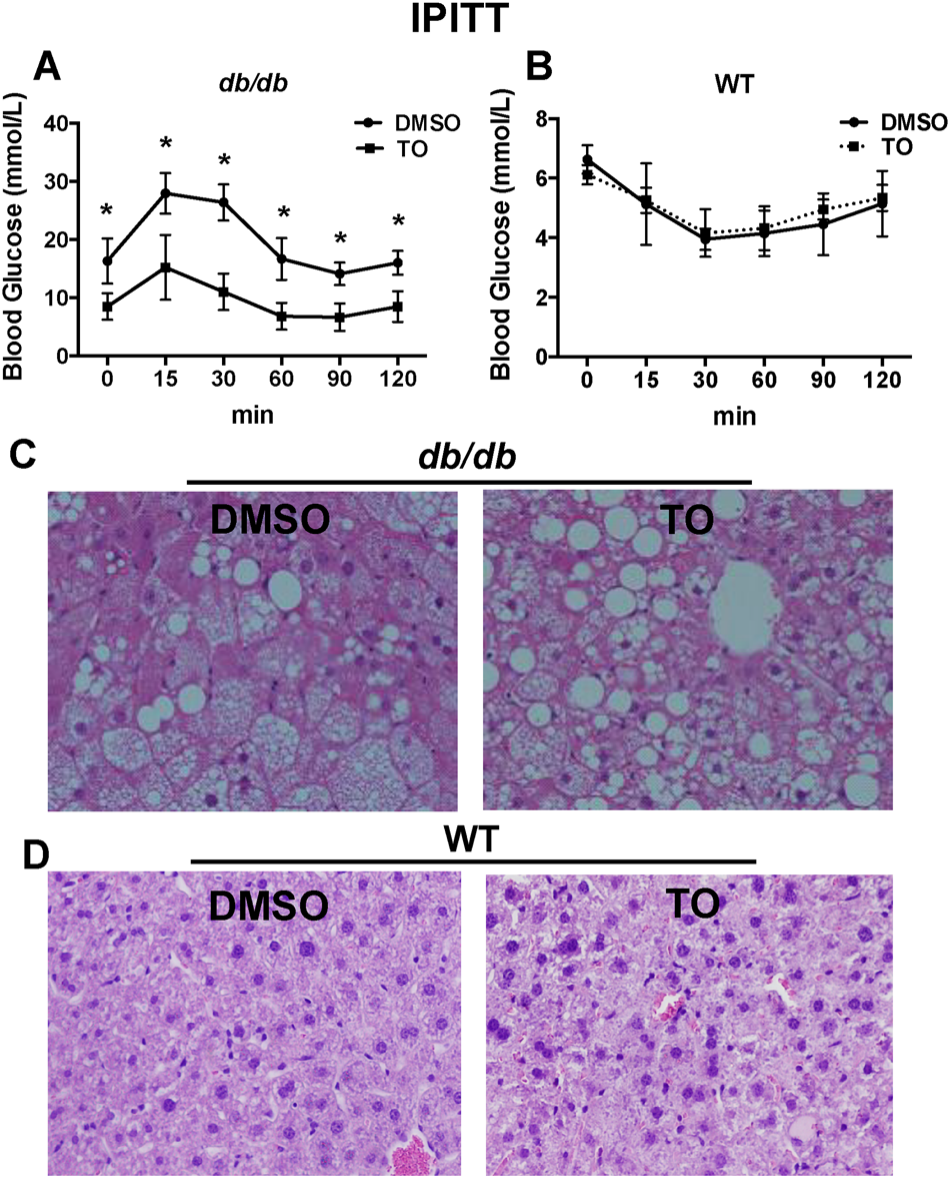
TO901317 (TO) improves insulin sensitivity in *db/db* mice but also increases the lipid accumulation in the liver. IPITT were performed on WT and *db/db* mice following 14 days of treatment with TO or DMSO (controls). 5 hours after last TO injection on the morning of day 14, mice already fasted for 12 hours were subjected to intraperitoneal insulin challenge (0.75 U/kg for WT and 1.5 U/kg for *db/db*). IPITT curves showed significant differences between the TO and the DMSO-treated *db/db* mice **(A)**, but no significant differences between the TO and the DMSO-treated WT mice **(B)**. H&E staining of the liver showed TO treatment increased the lipid accumulation, compared with the DMSO controls in *db/db* mice **(C)**, but no significant difference was observed in WT mice (**D**). Results in (**A**) and (**B**) are presented as mean ± SD, *P<0.05. 10–15 mice were used in each group.

At the fasting state, the blood glucose level is a reflection of hepatic glucose production, and thus HOMA-IR reflects primarily the liver insulin resistance. Therefore, IPITT was performed to investigate the effect of TO on the hypoglycemic response to insulin to further confirm if the whole body IR could be improved. As shown in Fig 2A and 2B, 2-week treatment with TO resulted in significantly lower levels of glucose in *db/db* mice, but not in the WT mice, at every time point of the IPITT when compared to the glucose levels of the control group.

As a characteristic of obese and diabetic model, *db/db* mice exhibited remarkable hepatic steatosis, which was worsened after TO treatment (Fig 2C and 2D). Similarly, TO treatment also exacerbated levels of hepatic TG and FFA (S2 Fig). No change in hepatic steatosis, TG or FFA was observed in TO-treated WT mice. These results suggest TO improves the glucose tolerance and insulin sensitivity only in *db/db* mice, but not WT mice.

### LXR agonist TO901317 suppressed the hepatic PEPCK, G-6-Pase expression, and activated Akt signaling pathway in *db/db* mice

**Fig 3:**
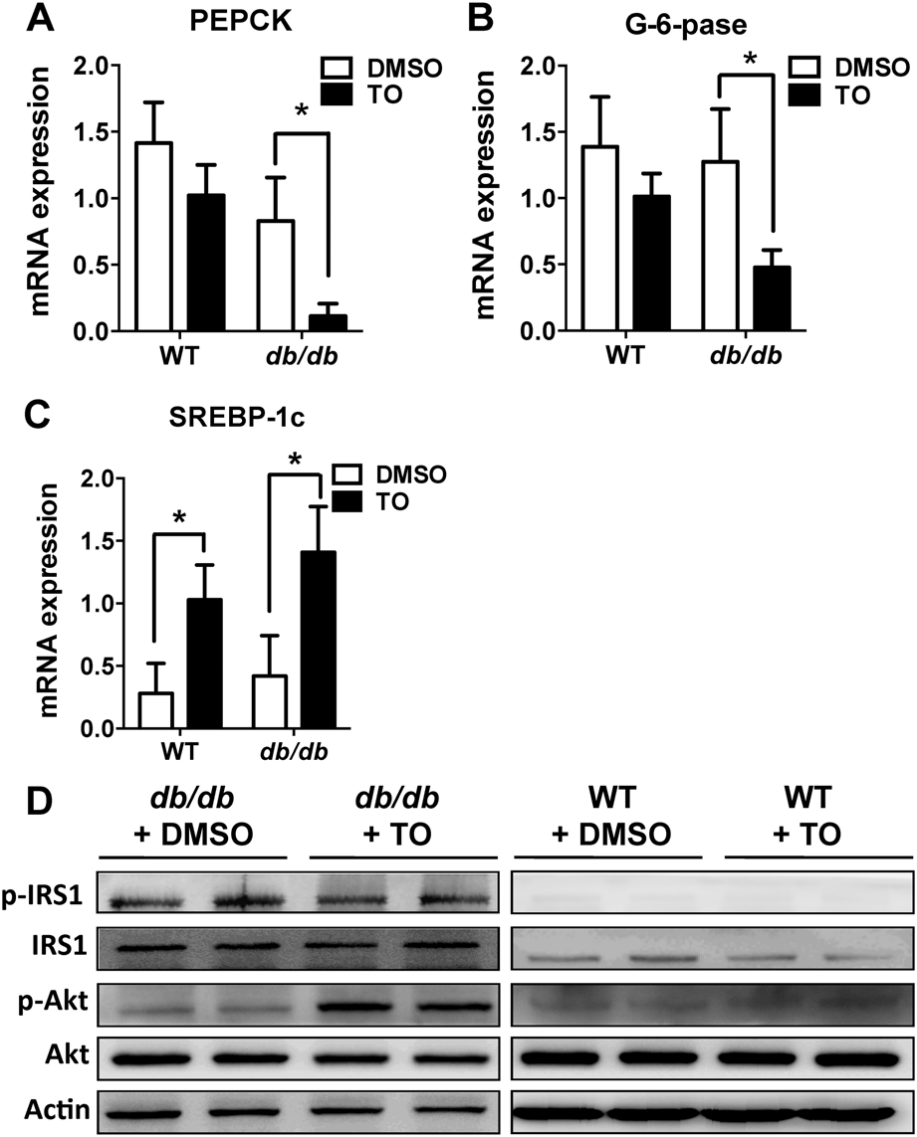
TO901317 (TO) suppressed the expressions of PEPCK and G-6-Pase and increased the Akt phosphorylation in *db/db* mice. **(A&B)** Expressions of PEPCK **(A)** and G-6-Pase **(B)** were significantly decreased in TO-treated *db/db* mice, compared with the DMSO-treated controls, but no significant differences between TO and DMSO-treated WT mice. **(C)** SREBP-1c expression was significantly increased in both WT and TO-treated *db/db* mice, compared with the respective DMSO-treated controls. **(D)** Insulin-stimulated phosphorylation of Akt was significantly increased in TO-treated *db/db* mice while the total Akt protein levels remained the same, but there was no change in either phosphorylation or total protein of IRS1, compared with DMSO-treated controls. TO had no effect on the phosphorylation and total protein of either Akt or IRS1 in WT mice. Results in (**A**), (**B**), and (**C**) are presented as mean ± SD, *P<0.05. 10–15 mice were used in each group.

Hepatic gluconeogenesis is one of the most important processes in regulating glucose metabolism. It is known that increased hepatic gluconeogenesis is due to overexpression of phosphoenolpyruvate carboxykinase (PEPCK) and glucose-6-phosphatase (G-6-pase), two key gluconeogenic enzymes. In assessing potential mechanisms underlying such an obvious improvement in glucose metabolism and insulin sensitivity in *db/db* mice, the mRNA expressions of PEPCK and G-6-pase in the liver were examined. We found PEPCK and G-6-pase expressions were significantly inhibited in TO-treated *db/db* mice, compared with DMSO-treated *db/db* mice (Fig 3A and 3B). As a positive control in the same experiment, SREBP-1c, as a known direct target gene of LXRs, was significantly increased in both TO-treated mice (*db/db* and WT), compared with the DMSO-treated mice (Fig 3C). Furthermore, insulin-stimulated phosphorylation of Akt (P-Akt) was significantly increased in TO-treated *db/db* mice, while the total Akt protein levels remained the same (Fig 3D). In contrast, the phosphorylation and total protein of IRS1 did not change in *db/db* mice. Meanwhile, the phosphorylation and total protein of neither Akt nor IRS1 were changed in WT mice.

### LXR agonist TO901317 suppressed oxidative stress (generation of ROS and MDA) and JNK signaling pathways in *db/db* mice

**Fig 4:**
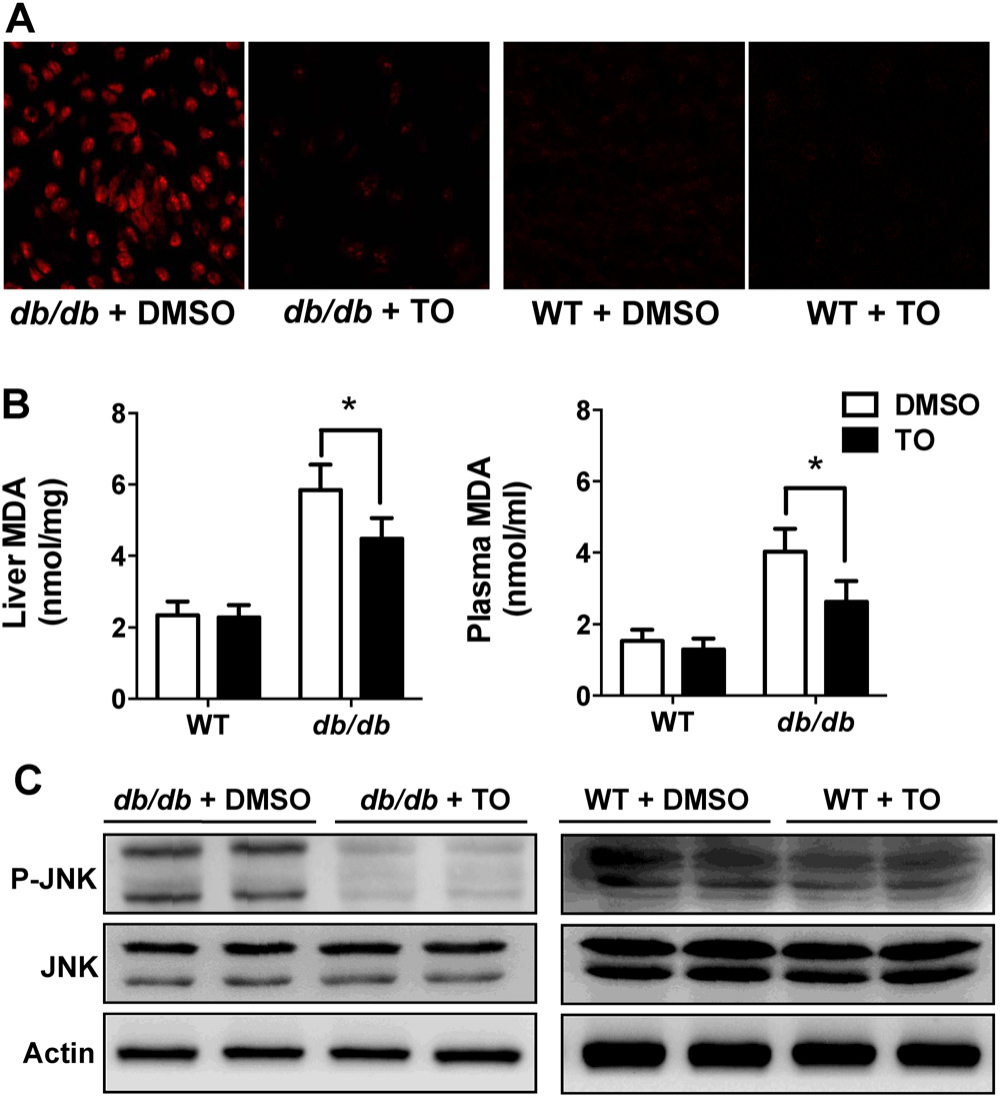
TO901317 (TO) suppressed both ROS production and JNK phosphorylation in the liver of *db/db* mice. **(A)** After 2 weeks treatment of TO or DMSO (control), ROS production in the liver was assessed by confocal microscope with *in situ* DHE stain. ROS generation was dramatically reduced in the TO-treated *db/db* mice, compared with that in DMSO controls. But there were no difference between the WT mice. **(B)** TO treatment (2 weeks) significantly reduced MDA levels in both plasma and liver of *db/db* mice, but not WT mice. **(C)** After 2 weeks treatment of TO or DMSO, mice liver tissues were collected for the WB analyses of JNK. JNK phosphorylation was significantly decreased in *db/db* mice with TO treatment, compared with that in DMSO controls, while the levels of total JNK protein were unaltered. No significant change was observed in either phosphorylation or total JNK protein levels in WT mice. Results in (**B**) are presented as mean ± SD, *P<0.05. Representatives of 10–15 mice in each group were shown.

In order to elucidate the mechanism how LXRs participate in the process of gluconeogenesis and why this phenomenon occurs only in the diabetic models with insulin resistance (not in the WT), we further investigated the relationship between the insulin pathway and LXRs. It has been reported that the oxidative stress plays the key role in insulin resistance [29]. Attenuation of excessive ROS generation can recover the insulin sensitivity, partly through suppressing the JNK activation [21]. Thus, we hypothesized that TO might alleviate insulin resistance via repressing the ROS production and JNK activation. Indeed, ROS production was dramatically reduced in the liver of TO-treated *db/db* mice when compared with that in DMSO-treated controls, (Fig 4A, **left panel**). The ROS level was very low (barely detectable) in the liver of WT mice, with either DMSO or TO treatment (Fig 4A, **right panel**). MDA formation, a reliable measure of lipid peroxidation [30], was also analyzed, from both blood and liver tissues, by thiobarbituric acid reactive substances (TBARS) assays (Fig 4B). Both plasma and hepatic levels of MDA were elevated in *db/db* mice relative to those of WT mice, and this elevated MDA in *db/db* mice was reduced significantly after TO-treatment. TO had no effect on the MDA levels in WT mice (Fig 4B).

Furthermore, JNK phosphorylation was significantly decreased in *db/db* mice with TO treatment, compared to that in the DMSO-treated controls, while the levels of total JNK protein were unaltered (Fig 4C, **left panel**). No significant change was observed in either phosphorylated or total JNK protein levels in WT mice regardless the treatment (Fig 4C, **right panel**). These results suggest the inhibition of oxidative stress by TO results in the suppression of the JNK activation, which might be one of the mechanisms leading to the improvement in blood glucose and the insulin resistance in *db/db* mice.

### Activation of LXRs by TO901317 in HepG2 cells restores palmitate-induced impairment of the insulin-signaling pathway, including both activation of Akt and the expression levels of PEPCK and G-6-Pase

**Fig 5:**
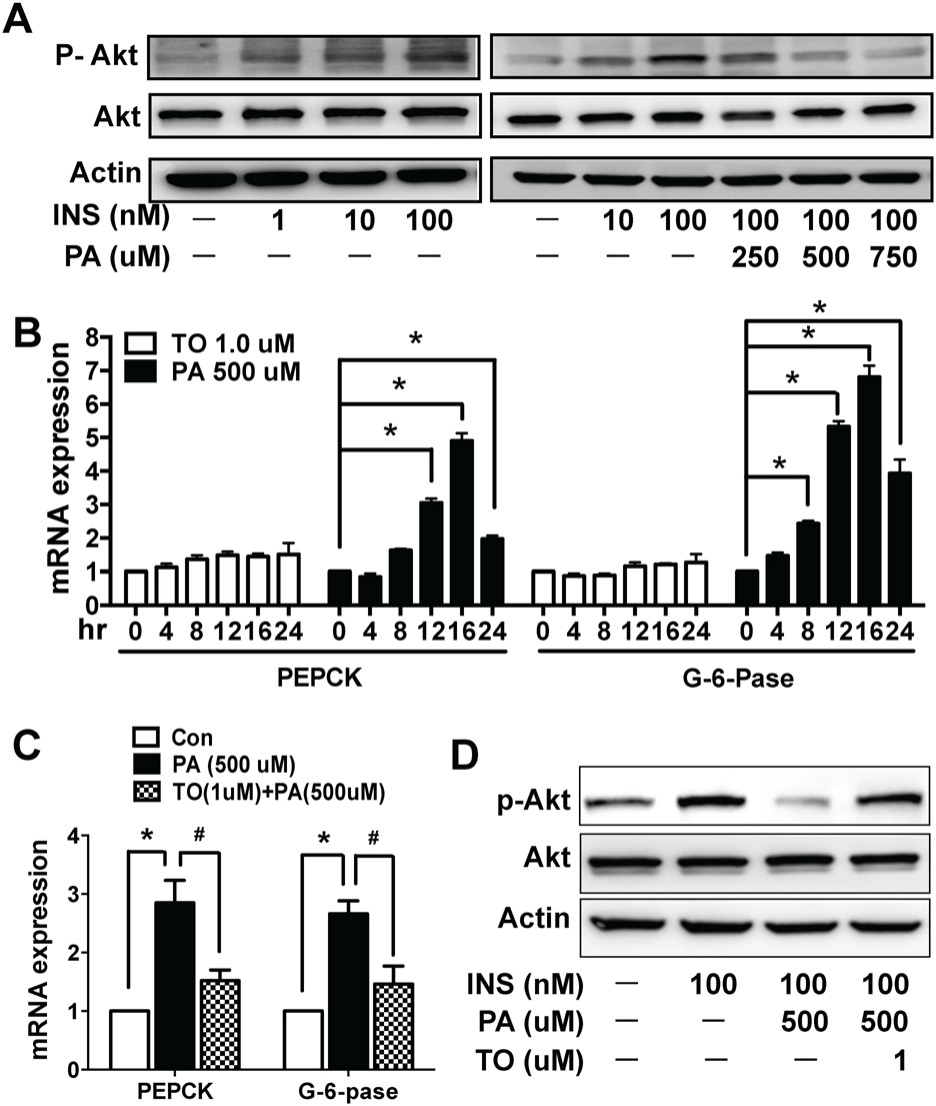
Effect of TO901317 on palmitate-induced alterations in insulin-stimulated phosphorylation of AKT and the expressions of PEPCK and G-6-Pase in HepG2 cells. **(A)** HepG2 cells were incubated in the presence or absence of palmitate (PA) [0 (control), 100, 250, 500, 750 μM] for 16 hr prior to 15 min stimulation with insulin (0, 1, 10, 100 nM). Total cell lysates were collected for WB analyses. Insulin activated Akt phosphorylation in HepG2 cells with a dose-dependent manner, but it was effectively suppressed by PA in a dose dependent manner, with almost a completely suppression at 750 μM. **(B)** HepG2 cells were incubated with either TO (1.0 μM) or PA (500 μM) for various periods of time (0, 4, 8, 12, 16, 24 hr) respectively. Total cell lysates were collected for the real-time PCR. PA treatment increased the expressions of PEPCK and G-6-Pase gradually over time, and peaked at 16hr, while there was no significant change in TO-treated cells. **(C &D)** HepG2 cells were incubated in the presence or absence of PA (500 μM) or PA (500 μM) + TO (1.0 μM) for 16 hr. Total cell lysates were collected for the real-time PCR and WB analyses. TO potently suppressed PA-stimulated expression of PEPCK and G-6-Pase **(C)**. Furthermore, TO reversed PA-suppressed Akt activation/phosphorylation without affecting total Akt expression **(D)**. Results in (**B**) &(**C**) are presented as mean ± SD, *^, #^P<0.05. 10–15 mice were used in each group.

To further explore the molecular mechanisms how FFA is involved in regulating insulin signaling, we chose HepG2 cells, one of the most widely used hepatic cell line, as a cell culture model. Palmitate (PA), a known FFA widely used in insulin resistance research, was used to treat HepG2 cells. First, we demonstrated that the insulin activated Akt in HepG2 cells, measured as Akt phosphorylation, in a dose-dependent manner (Fig 5A, **left panel**). The insulin-stimulated activation of Akt was effectively suppressed by PA in a dose dependent manner, with almost a completely suppression at 750 μM (Fig 5A, **right panel**). We further examined whether PA affected the transcriptional expressions of PEPCK and G-6-Pase. As shown in Fig 5B, PA treatment increased the expressions of PEPCK and G-6-Pase gradually over a few hours, and peaked at 16 hr, the same time point when PA maximally suppressed insulin-stimulated Akt phosphorylation. No change was observed in the expression of the two genes in the presence of 1.0 μM TO, as shown in Fig 5B. These results indicate that PA impairs the insulin-signaling pathway and suppresses the expressions of PEPCK and G-6-Pase, which might ultimately resulted in abnormal glucose metabolism. To determine if TO could reverse the effects of PA stimulation in HepG2 cells, TO (1.0 μM) was co-incubated with PA (500 μM) for 16hr. As shown in Fig 5C, TO potently suppressed PA-stimulated expression of PEPCK and G-6-Pase. Furthermore, TO reversed PA-suppressed Akt phosphorylation without affecting Akt expression (Fig 5D). Our *in vitro* data with HepG2 cell model are consistent with what we have observed *in vivo* in mice in that activation of LXRs by TO can effectively improve insulin resistance and the glucose metabolism.

### LXR agonist TO901317 suppressed the ROS production and JNK activation in palmitate-treated HepG2 cells

**Fig 6:**
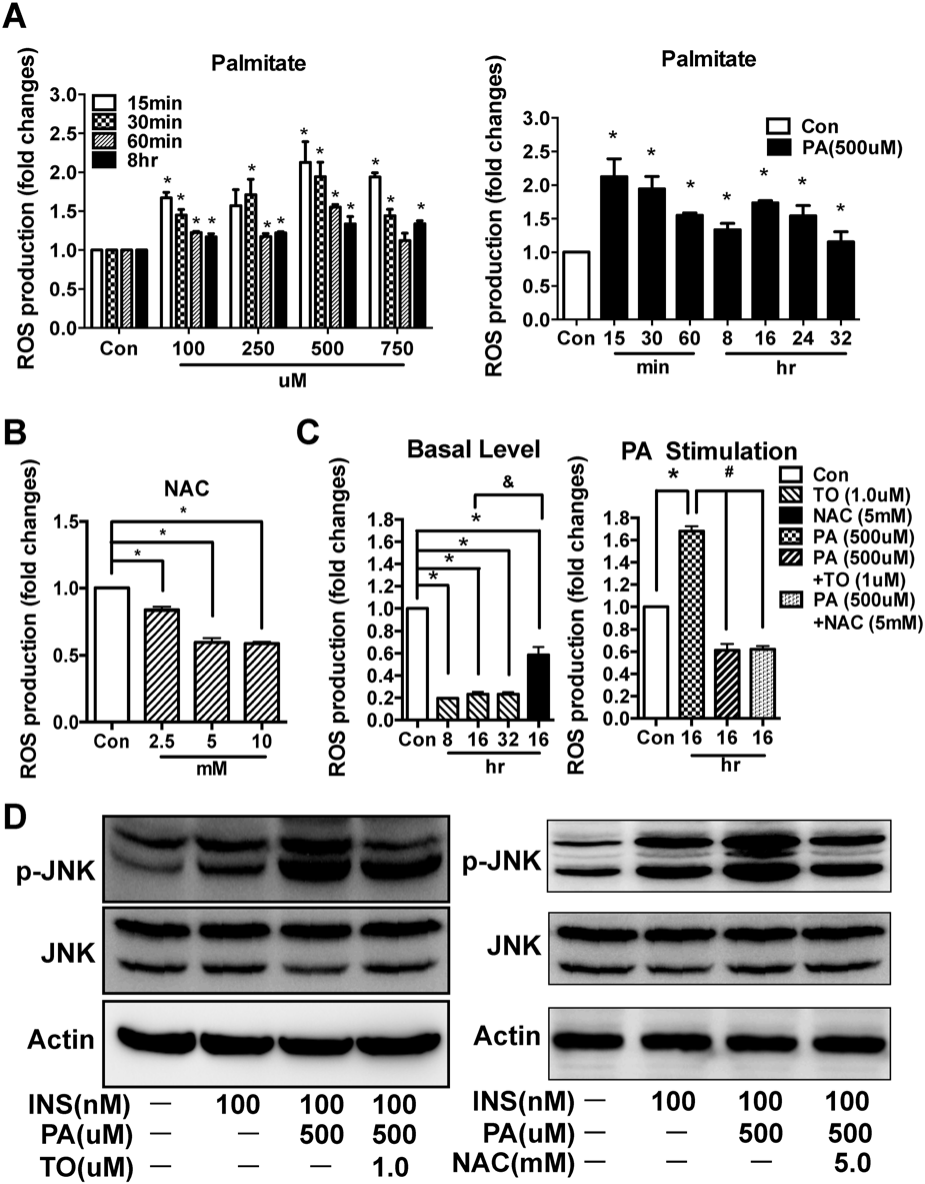
TO901317 (TO) inhibited the ROS production and JNK phosphorylation in Palmitate-treated HepG2 cells. **(A)** Left panel: HepG2 cells were incubated with palmitate (PA) in various doses [100, 250, 500 and 750 μM; control (no treatment)] for different periods of times (15, 30, 60 minutes, and 8 hr), respectively. Intracellular ROS production was quantified using the fluorescent probe DCFH-DA. PA treatment increased the ROS production in a dose dependent manner when concentration is 500 μM or lower; and this elevation was sustained for up to 8 hr. Right panel: Since 500 μM of PA was most potent in inducing ROS production as shown in the left panel, we further tested if PA (500 μM) has prolonged effects (at 8, 16, 24, 32 hr time points). Indeed, the PA stimulated ROS sustained for up to 32 hr. **(B)** HepG2 cells were incubated for 16hr with increasing concentrations of NAC (0, 2.5, 5,10 mM), a positive control for ROS suppression. NAC treatment decreased ROS production in a concentration dependent manner. **(C)** HepG2 cells were either incubated with TO (1.0 μM) for 8, 16, 32 hr or with 5 mM NAC for 16 hr (Left panel); or incubated for 16 hr with PA (500 μM), PA (500 μM) + TO (1.0 μM), and PA (500 μM) + NAC (5 mM), respectively (Right panel). Under basal (unstimulated condition) (Left panel), TO markedly suppressed the ROS production, with potency higher than NAC. Under PA-stimulated condition (Right panel), TO treatment (for 16 hr) had a similar potency as NAC (16 hr) in suppressing ROS production. **(D)** Activation of JNK was analyzed by SDS-PAGE and Western blot. Both TO (left panel) and NAC (right panel) suppressed the PA-induced JNK activation/phosphorylation in HepG2 cells. Results in (**A**), (**B**), and (**C**) are presented as mean ± SD, *P<0.05 versus control, ^&^P<0.05 NAC versus TO, ^#^P<0.05 versus PA.

To further explore the relationships between LXR, ROS, and JNK pathway, we tested the effects of TO on PA-stimulated ROS production and JNK activation in HepG2 cells. In the absence of TO, PA treatment increased the ROS production, both acutely (15–60 min) and chronically (8 hr), in a dose dependent manner, with the peak at 500 μM (Fig 6A, **left panel**). We further tested if PA (500 μM) has even more prolonged effects beyond 8 hr (at 16, 24, 32 hr time points). Indeed, while the chronically stimulated ROS by PA was peaked at 16 hr, it sustained for up to 32 hr (Fig 6A, **right panel**). Since PA at 500 μM was most potent in inducing ROS production (Fig 6A, **left panel**), 500 μM of PA at 16 hr of incubation time were chosen for most of our studies using HepG2 cells. NAC, a classic antioxidant, was chosen as a positive control for the suppression of ROS. In the presence of increasing concentrations of NAC for 16 hr, ROS production decreased in a concentration-dependent manner, with the maximum effect of NAC at 5 mM [higher concentration (10 mM) had similar effect as 5 mM] (Fig 6B). Therefore, 5 mM NAC was chosen for the follow-up experiments. When comparing the effects of TO and NAC at steady state (Fig 6C, **left panel**), TO reduced the ROS production by ~5-fold than the control, almost 3-fold more potent than NAC (only ~40% ROS suppression), and lasted as long as 32 hr. Moreover, TO also effectively suppressed PA-simulated ROS production, with a similar potency of NAC (Fig 6C, **right panel**). These results indicate that the anti-oxidative effect of TO901317 is both potent and persistent.

To confirm the correlation between TO-mediated ROS suppression and JNK inactivation we identified in *db/db* mice (Fig 4), the effects of TO on JNK phosphorylation was tested in HepG2 cells. As expected, the JNK phosphorylation was markedly enhanced by PA, and this enhancement was diminished in the presence of TO (Fig 6D, **left panel**). We further determined whether NAC, as an antioxidant, had a similar effect as TO on activation/phosphorylation of JNK in HepG2 cells. 5 mM NAC were incubated with HepG2 cells for 16 hr, in the absence or presence of 500 μM PA. As we expected, NAC had similar effect as TO on the phosphorylation of JNK (compare Fig 6D, **left and right panels**).

### TO-treated *db/db* mice and HepG2 cells express higher levels of a major hepatic anti-oxidative gene Nrf2 than control counterparts

Since TO potently suppressed both ROS production *in vivo* (*db/db* mice; Fig 4) and *in vitro* (HepG2 cells; Fig 6), we further determine if TO treatment alters anti-oxidative genes in both *db/db* mice and HepG2 cells. As shown in S4A Fig, Nrf2, Mn-SOD, two of the 8 major anti-oxidative genes tested, were elevated in TO-treated *db/db* mice compared to those in DMSO-treated control *db/db* mice. The Nrf2 expression also increased in TO-treated HepG2 cells compared to that of control (S4B Fig).

## Discussion

The current study was designed to explore the mechanism of the antidiabetic effect of LXRs. We demonstrated that TO901317, a highly specific LXRs agonist, potently improved the hepatic glucose metabolism by activating Akt signaling pathway. We also showed that the activation of hepatic LXRs led to inhibition of ROS production and inactivation of JNK pathway. Our data strongly support a beneficial role of LXR activation in improving insulin resistance and suggest LXRs as a potential therapeutic target for treating type 2 diabetes.

Obesity is associated with a rising risk of developing insulin resistance and type 2 diabetes [31]. In this study, *db/db* mice were chosen as the obesity-induced insulin resistance and diabetic mouse model. First, in *db/db* mice, we found LXR agonist TO potently suppressed the obvious diabetic phenotypes, including a decrease in the levels of fasting glucose, fasting insulin and HOMA-IR. Our IPITT data further indicated a great improvement of insulin sensitivity in TO-treated *db/db* mice without the risks of hypoglycemia. An interesting aspect of LXR-mediated glucose and insulin regulation is that this regulation occurs only in *db/db* mice but not in WT mice. Unlike *db/db* mice, WT mice exhibited no significant change in plasma glucose, insulin level, HOMA-IR and IPITT after TO treatment. Similar phenomenon was reported previously in various rodent models [5, 11–14]. There was also no statistical difference in average food consumption between the TO-treated animals and the control groups (S1 Fig). This would suggest that the TO-mediated glucose metabolic improvement was not due to its effects on the food consumption or body weight. These data indicates an effective improvement of glucose and insulin sensitivity by LXR agonist occurs only in IR obese rodents, not in normal mice.

Previous studies indicated LXRs activation would cause hepatic lipogenesis through direct activation of sterol responsive element-binding protein-1c (SREBP-1c) transcription [32,33]. In our study, TO-treated *db/db* mice exhibited more extensive hepatic steatosis than the control group, most likely due to SREBP-1c-mediated up-regulation of lipogenetic genes, such as SCD-1 and FAS, etc [32]. Hepatic steatosis usually led to aggravation of IR. Interestingly however, this phenomenon did not happen in our TO-treated *db/db* mice. Instead, the TO-treated *db/db* mice exhibited significant improvement of blood glucose and insulin, as well as IR. Recently, Gustafsson group reported that GW3965, another LXR agonist, increased intrahepatic TG level but reduced serum TG level through the activation of serum lipase activity in chow diet-fed mice with 5 weeks treatment [34]. There are also examples of steatosis without being associated with hepatic insulin resistance; steatosis occurs even when insulin sensitivity is dramatically improved [35,36]. Similar results were observed in high fat diet-induced obesity model with WT mice. Gao and Liu [37] demonstrated that 10-week TO treatments could protect WT mice from high fat diet-induced obesity and obesity-associated insulin resistance and glucose impairment, while withdrawal of TO could reverse the steatosis in the liver. Thus, although activation of LXRs increased lipogenesis, it does not affect its role in the improvement of hyperglycemia and insulin sensitivity.

Insulin exerts two main actions in liver: gluconeogenesis and lipogenesis. These processes are attributable, at least in part, to insulin—induced phosphorylation of the down-stream signaling molecules and the regulation of associated genes. In the IR state, insulin is unable to inhibit gluconeogenesis but still retains its ability to enhance lipogenesis, which is called “selective insulin resistance” [38]. Because of unsuppressed gluconeogenesis, part of the exceeded blood glucose might be used as a substrate participating the hepatic lipogenesis, which will further aggravate the IR and lead to the vicious cycle in glucose and lipid metabolism. However, such a vicious cycle seemed to be interrupted by activated LXRs. In the liver of TO-treated *db/db* mice, the transcriptional repression of PEPCK and G-6-Pase was more than 85%, and 60%, respectively, with a markedly increased activation (phosphorylation) of Akt. The enhanced phosphorylation of Akt via LXRs might be the ultimate reason of the improved diabetic phenotype in TO-treated *db/db* mice, even when the liver steatosis was increased. Our data strongly indicated the TO-activated LXRs acted similarly like insulin: promoting the insulin signaling pathway and suppressing the gluconeogenetic genes. The precise mechanism of such a dramatic inhibition of these two key gluconeogenesis genes was not known, as Stulnig has reported that PEPCK and G-6-Pase are not the direct target genes of LXRs [39]. We provide a novel finding in that activation of LXRs by TO leads to the activation of Akt, resulting in improved insulin sensitivity in *db/db* mice. On the other hand, LXRs activation has no effect on activation of IRS1, another pivotal protein involved in insulin pathway, suggesting that LXRs either by-pass IRS and directly activate insulin signaling pathway, or only partially regulate insulin signaling pathway. The fact that TO does not affect gluconeogenesis and Akt activation in WT mice suggests the hepatic activation of LXRs regulates insulin-signaling pathway only under IR condition and the up-regulated SREBP-1c expression does not induce the lipogenesis in the liver of WT mice. The reason for the opposite effects of TO on liver steatosis and IR is not fully understood, and needs further investigation.

It is necessary to point out that, since LXR activation is known to stimulate lipogenesis as we mentioned earlier, we cannot exclude the potential possibility that TO-treatment may facilitate the conversion of extra glucose to lipid and store as triglyceride (TG) in the liver, thereby as one of the mechanisms contributing to the reduction of hyperglycemia in *db/db* mice.

The mechanism of actions by activated LXRs was further investigated in a cell model using HepG2 cells. Since *db/db* mice had high levels of FFAs with IR, we considered FFAs might be one of the main factors inducing IR. Plasma FFAs are composed of the saturated fatty acid palmitate (about 30–35%) and of the mono-unsaturated fatty acid oleate (about 40–50%). A series of recent clinical studies indicated insulin resistance and type 2 diabetes were closely associated with relatively high intake of saturated fat (e.g. palmitic acid) [40]. We therefore chose Palmitate (PA) as the inducer to establish the IR cell model. Based on cell toxicity data of ours (S3 Fig) and others’ [41,42], PA treatment at 500 μM for 16 hr could potently induce IR through specific impairment of insulin signaling pathway while exhibiting little cytotoxicity. In HepG2 cells, while insulin-mediated Akt activation was inhibited by PA in a dose-dependent manner, PEPCK and G-6-Pase expressions were increased greatly by PA after 8 hr treatment and lasted to 24 hr. These results indicated the insulin signaling had been impaired in the PA-stimulated HepG2 cells. The effects of PA on Akt, PEPCK, and G-6-Pase were effectively reversed by TO. These *in vitro* data were consistent with what we found in *db/db* mice: The impaired insulin signaling can be reversed by LXR activation, leading to Akt activation and the gluconeogenesis suppression, which happened only under IR state.

It has been suggested that increased reactive oxygen species (ROS) levels are an important trigger for insulin resistance [43]. Also, oxidative stress has been proposed as a link between FFA and hepatic insulin resistance [44,45]. Recently, a series of clinic studies demonstrated that patients with metabolic disorders could benefit from anti-oxidative treatments through monitoring some oxidative stress biomarkers in blood and tissues [46,47]. Thus, we investigated the changes of ROS levels in our mice model. Compared with the WT mice, *db/db* mice had a significantly higher level of hepatic ROS and oxidative production (MDA) in both blood and liver. TO treatment potently suppressed the ROS production and MDA level in *db/db* mice, accompanied by a dramatic inhibition of JNK activation and activation of Akt, but had no effect on those of WT mice. Similar ROS production results were also observed in our HepG2 cell model: PA treatment increased ROS production and JNK phosphorylation while decreased Akt activation, suggesting that, in the presence of excess ROS, JNK pathway was activated and Akt activation was impaired. These effects of PA could be potently reversed by activation of LXRs using TO.

To determine if the TO-mediated activation of Akt and suppression of JNK is a direct consequence of anti-oxidative effects, we tested if N-acetyl-cysteine (NAC), a classic antioxidant and widely used for reducing oxidative stress [48], had a similar effect on JNK and Akt as TO. NAC treatment resulted in effective suppression of ROS production and JNK activation, strikingly similar to the effects of TO901317. Interestingly, when comparing the anti-oxidative effects of TO901317 with NAC, we found TO901317 was more potent. These results further verified that anti-oxidative treatment could improve IR.

How does TO exert anti-oxidative effects? There is so far no report indicating that TO has any ability or property to directly act on the ROS, although we cannot exclude the possibility that TO may directly induced antioxidative gene such as Nrf2. Based on the current literature, we hypothesized that TO901317 might suppress ROS generation through LXR activation, which up-regulated the expression of anti-oxidative genes. Indeed, we found an increased expression of two anti-oxidative genes in TO-treated *db/db* mice, including Mn-SOD (Mn-superoxide dismutase) and NF-E2 related factor 2 (Nrf2), compared with *db/db* control mice (S4 Fig; **left panel**). Increased Nrf2 expression was also observed in TO-treated HepG2 cells (S4 Fig; **right panel**). Therefore, it is likely that Nrf2 is one of the target genes of TO-mediated LXR activation, leading to an enhanced ROS scavenging, although the underlying mechanism remains to be explored.

Nrf2, a nuclear transcriptional factor, functions in the transcriptional activation of several antioxidant defense genes such as HO-1, Prx I, and SOD through binding to their antioxidant response element (ARE) sequences. Nrf2 pathway has been demonstrated to play a critical role in anti-oxidative stress [49, 50]. Since oxidative stress is one of the main inducers of insulin resistance, it is conceivable that Nrf2 may play a regulatory role in insulin signaling pathway. Nrf2 pathway activation or induced expression of Nrf2 has been demonstrated to have significant effects on the improvement of glucose homeostasis and insulin sensitivity *in vivo* and *in vitro* [51,52]. To our knowledge, there’s no report on the relationship between TO and the regulation of antioxidant genes in the liver, either directly or indirectly. However, there are studies on the function of LXRs in anti-oxidative stress in other organs. One report demonstrated that LXRα-KI (knock-in) mice induced elevated expression of several antioxidant enzymes and a decreased production of ROS in the lung [53]. Another report showed LXRs activation alleviated high glucose-induced oxidative stress in endothelial progenitor cells through AMPK pathway [26]. Most recently, it was demonstrated that the activation of LXRs by GW3965 (another LXR agonist) attenuated the cardiac dysfunctions of db/db mice and the redox disorder in mice with myocardial ischemia/reperfusion injury, partly through relieving the oxidative stress via regulating another key anti-oxidase gp^91phox^[54,55]. All these data at least in part support our hypothesis. On the other hand, since LXRs are known to inhibit inflammatory responses, we cannot exclude the possibility that LXR-mediated regulation of cytokine expression was indirectly involved in the regulation of antioxidant genes. Therefore, while our data may provide a preliminary basis for potentially new therapeutic strategies through manipulating LXRs activation, the exact cause-and-effect relationship between the LXRs and anti-oxidative genes merits further investigation.

In summary, we present a novel molecular mechanism involved in LXR-mediated glucose metabolism. Our data demonstrate a new role of LXR activation in regulating oxidative stress response, which lead to a reduced hyperglycemia and an improved insulin resistance through at least in part the suppression of ROS production and JNK pathway and the activation of Akt. Our study provides novel evidence and new molecular mechanism to support LXRs as promising therapeutic targets for drug development in treating insulin resistance-associated obesity, diabetes, and the subsequent metabolic diseases.

## Supporting Information

S1 Fig TO901317 (TO) and DMSO (control) had no effect on the food intake of either WT or *db/db* mice. The average daily food intake of each mouse over 14 days was analyzed. There is no change between the TO and DMSO groups, either in WT or in *db/db* mice, although *db/db* mice consumed twice as much foods as the WT mice.
(PDF)

S2 Fig TO901317 (TO) induced the hepatic lipogenesis in *db/db* mice. The lipid panel of the liver was measured in both *db/db* and WT mice with or without TO treatment, using an assay kit according to the manufacturer’s instructions (Nanjing Jiancheng bioengineering research institute, China). TO treatment resulted in increases of both hepatic TG and FFA levels in *db/db* mice, but not WT mice, compared with DMSO controls. TO had no effect on TC levels in either WT or *db/db* mice. Results are presented as mean ± SD, *P<0.05. At least 10 mice in each group were used.
(PDF)

S3 Fig Cell viability assays (MTT assay) in HepG2 cells treated with Palmitate. PA treatment at 750 μM caused a significant inhibition in cell viability after 12 hours, while no significant change in cell viability at 500 μM PA till 24 hours. PA at 250 μM exhibited no effect on cell viability at any time point tested. The HepG2 cells were seeded in a 96-well plate (1×10^4^ cells/well) and then incubated with various concentrations of PA (0, 250, 500, 750 μM) and for different periods of time (8, 12, 16, 24 hours). MTT (Sigma) solution was added into each well and incubated for 4 hours at 37°C. DMSO was added into the wells (100 μl/well) and the resultant formazan product was measured at 490 nm using a VersaMax ELISA Microplate Reader (Molecular Devices). Each sample was triplicated, and experiments were repeated three times. Results are presented as mean ± SD, *P<0.05 versus controls.
(PDF)

S4 Fig TO901317 (TO) promoted expressions of anti-oxidative genes in both *db/db* mice and HepG2 cells. Expressions of several well-characterized anti-oxidative genes, as labeled, were analyzed in the liver of *db/db* mice after 2 weeks TO treatment (DMSO treatment as controls) or in TO-treated HepG2 cells (1.0 μM TO for 16 hr). Nrf2 and Mn-SOD expressions were increased by the TO treatment in *db/db* mice (A), while Nrf2, but not Mn-SOD, was enhanced in palmitate-treated HepG2 cells (B). Results are presented as mean ± SD, *P<0.05. Primers used in real-time PCR were showed in S1 Table.
(PDF)

S1 Table Sequences of the primers used in real time PCR. (PDF)
