# Supplementary figures and images for: Activation of the Liver X Receptor by Agonist TO901317 Improves Hepatic Insulin Resistance via Suppressing Reactive Oxygen Species and JNK Pathway
Source: PLoS One. 2015 Apr 24;10(4):e0124778. doi: 10.1371/journal.pone.0124778 (PMC4409387; doi:10.1371/journal.pone.0124778)

**S1 Fig**

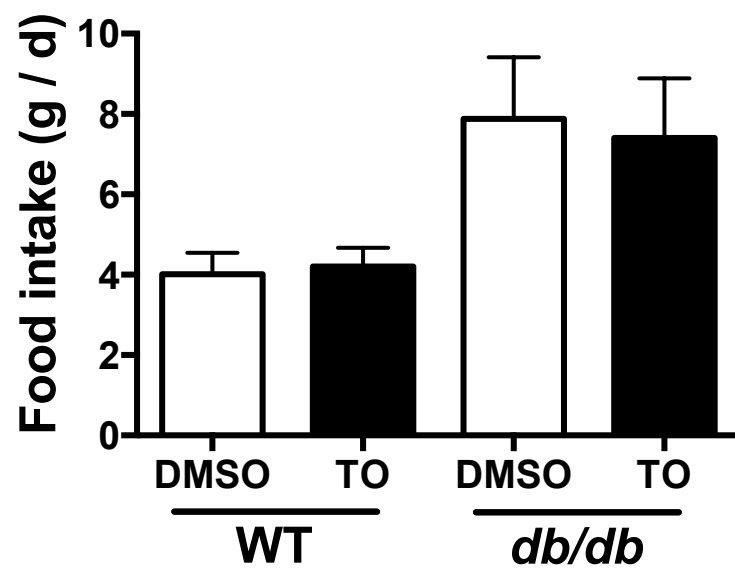

Supplement: S1 Fig — The average daily food intake of each mouse over 14 days was analyzed. There is no change between the TO and DMSO groups, either in WT or in db/db mice, although db/db mice consumed twice as much foods as the WT mice. (PDF) [file pone.0124778.s001.pdf]

S2 Fig

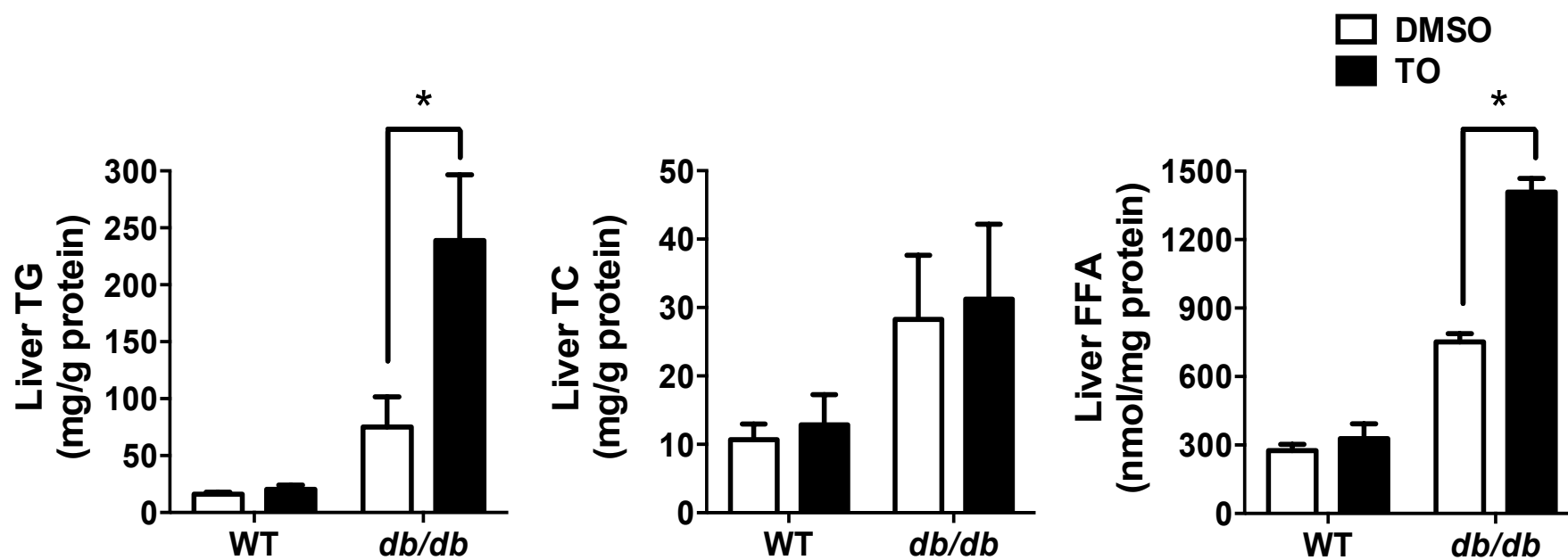

Supplement: S2 Fig — The lipid panel of the liver was measured in both db/db and WT mice with or without TO treatment, using an assay kit according to the manufacturer’s instructions (Nanjing Jiancheng bioengineering research institute, China). TO treatment resulted in increases of both hepatic TG and FFA levels in db/db mice, but not WT mice, compared with DMSO controls. TO had no effect on TC levels in either WT or db/db mice. Results are presented as mean ± SD, *P<0.05. At least 10 mice in each group were used. (PDF) [file pone.0124778.s002.pdf]

S3 Fig

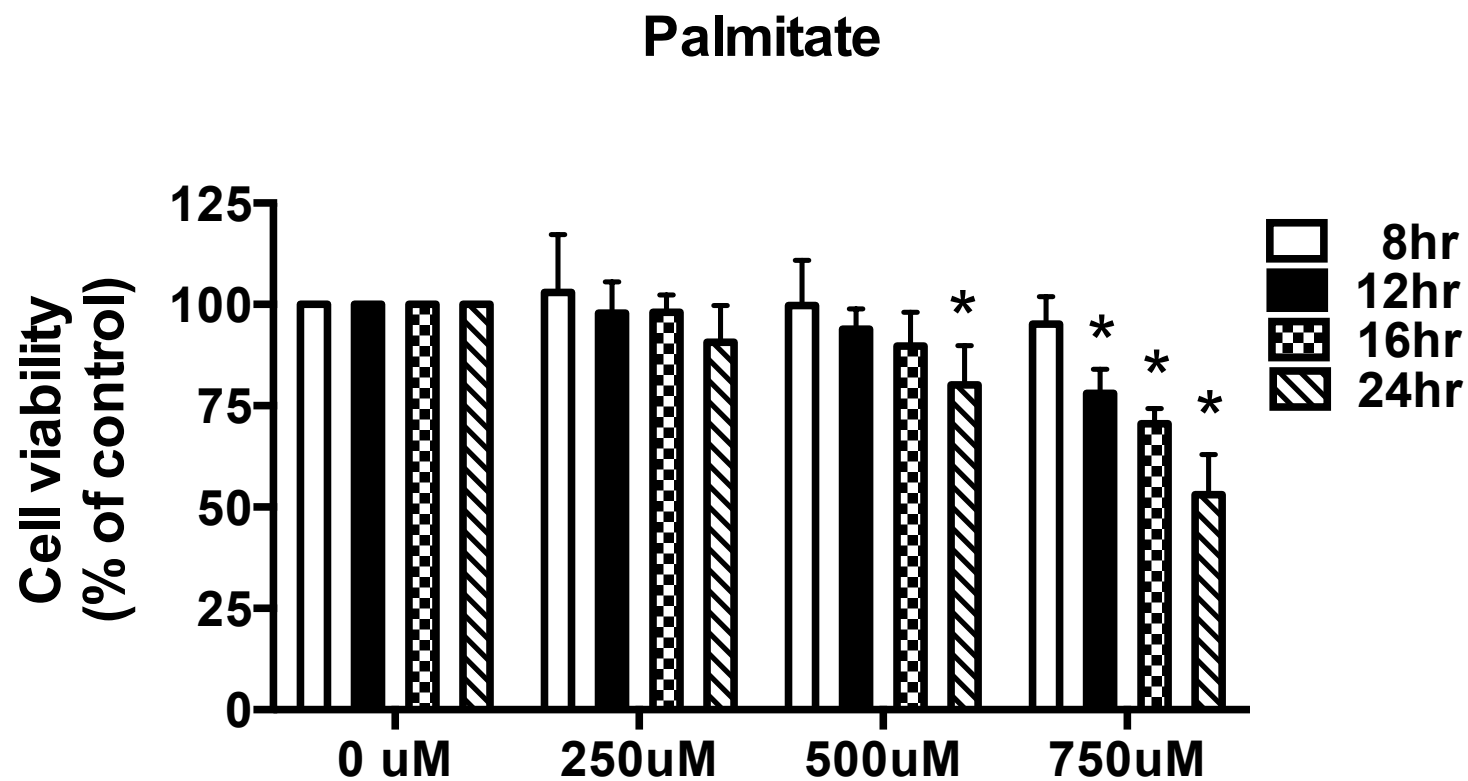

Supplement: S3 Fig — PA treatment at 750 μM caused a significant inhibition in cell viability after 12 hours, while no significant change in cell viability at 500 μM PA till 24 hours. PA at 250 μM exhibited no effect on cell viability at any time point tested. The HepG2 cells were seeded in a 96-well plate (1×10^4^ cells/well) and then incubated with various concentrations of PA (0, 250, 500, 750 μM) and for different periods of time (8, 12, 16, 24 hours). MTT (Sigma) solution was added into each well and incubated for 4 hours at 37°C. DMSO was added into the wells (100 μl/well) and the resultant formazan product was measured at 490 nm using a VersaMax ELISA Microplate Reader (Molecular Devices). Each sample was triplicated, and experiments were repeated three times. Results are presented as mean ± SD, *P<0.05 versus controls. (PDF) [file pone.0124778.s003.pdf]

S4 Fig

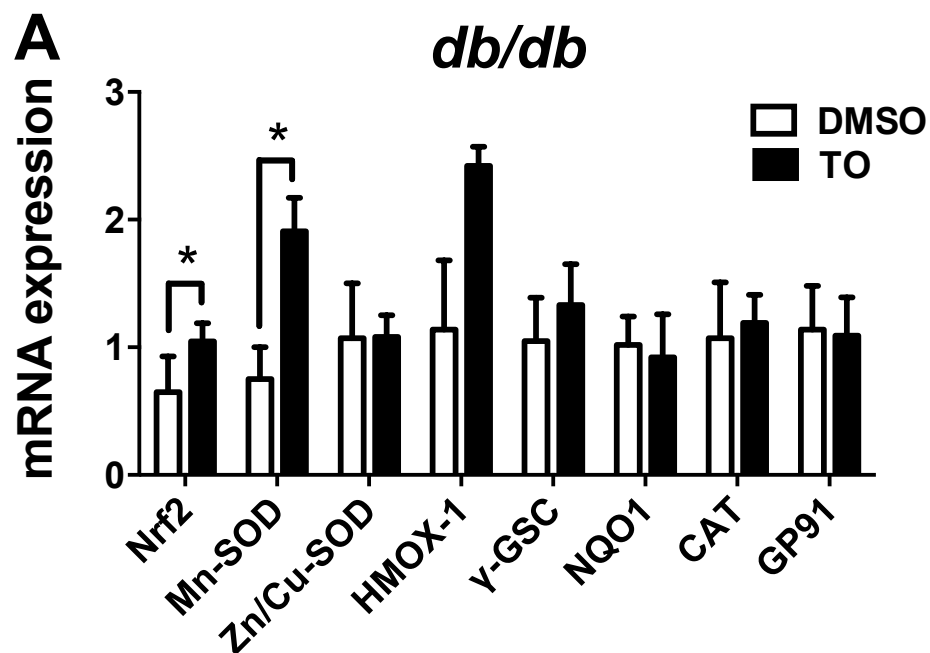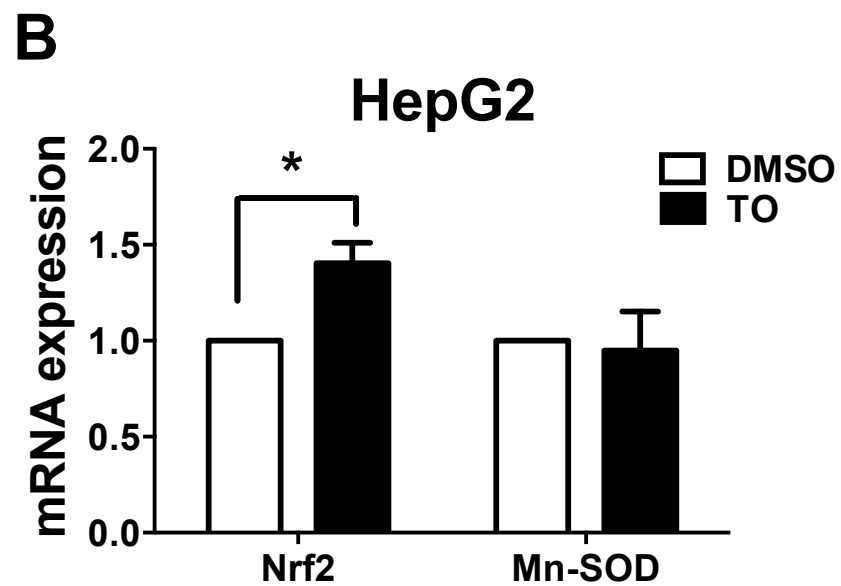

Supplement: S4 Fig — Expressions of several well-characterized anti-oxidative genes, as labeled, were analyzed in the liver of db/db mice after 2 weeks TO treatment (DMSO treatment as controls) or in TO-treated HepG2 cells (1.0 μM TO for 16 hr). Nrf2 and Mn-SOD expressions were increased by the TO treatment in db/db mice (A), while Nrf2, but not Mn-SOD, was enhanced in palmitate-treated HepG2 cells (B). Results are presented as mean ± SD, *P<0.05. Primers used in real-time PCR were showed in S1 Table. (PDF) [file pone.0124778.s004.pdf]
