# Supplementary material for: Activation of the Liver X Receptor by Agonist TO901317 Improves Hepatic Insulin Resistance via Suppressing Reactive Oxygen Species and JNK Pathway
Source: PLoS One. 2015 Apr 24;10(4):e0124778. doi: 10.1371/journal.pone.0124778 (PMC4409387; doi:10.1371/journal.pone.0124778)
Supplement: S1 Table — (PDF) [file pone.0124778.s005.pdf]

S1 Table. Sequences of the primers used in real-time PCR

GENES	SPECIES	FORWARD PRIMER	REVERSE PRIMER
**Nrf2**	Mouse	AGTCCCAGCAGGACATGGATT	AGTTGCTCTTGTCTTTCCTTTTCG
**Mn-SOD**	Mouse	GGCCAAGGGAGATGTTACAACTC	CCAAAGTCACGCTTGATAGCCT
**Zn/CU-SOD**	Mouse	TGCAGGGAACCATCCACTTC	AAATGAGGTCCTGCACTGGTACA
**HMOX-1**	Mouse	GTCTCGAGCATAGCCCGGA	TTCTCGGCTTGGATGTGTACCT
**γ-GCS**	Mouse	ATCTGCTTTGCTGTCGGTGC	CAATTTTGTCCCAACTTCAGGC
**NQO1**	Mouse	CTACGCCATGAAGGAGGCTG	CGGAAGGATACTGAAAGTTCTTCG
**CAT**	Mouse	GCAGCTCCGCAATCCTACAC	CCCCGCGGTCATGATATTAA
**GP91**	Mouse	TGATCCTGCTGCCAGTGTGTC	GTGAGGTTCCTGTCCAGTTGTCTTC
**β-actin**	Mouse	CCTTCTACAATGAGCTGCGTG	ACAGCCTGGATAGCAACGTAC
**Nrf2**	Human	GAAACCAGTGGATCTGCCAACT	TCGATGTGACCGGGAATATCA
**Mn-SOD**	Human	CCCGACCTGCCCTACGACTA	GATGGCTTCCAGCAACTCCC
**GAPDH**	Human	CCTTCTACAATGAGCTGCGTG	ACAGCCTGGATAGCAACGTAC
